# Wrist Circumference–Dependent Thresholds for the Median Nerve in Carpal Tunnel Syndrome: A Cross-Cohort Comparison

**DOI:** 10.1055/a-2837-4022

**Published:** 2026-04-30

**Authors:** Tom B. G. Olde Dubbelink, Federica Ginanneschi, Paolo Falsetti, Jan Meulstee, Ronald H. M. A. Bartels, Wim I. M. Verhagen

**Affiliations:** 1 Department of Neurology, Radboud University Medical Center, Donders Institute for Brain, Cognition, and Behavior, Nijmegen, the Netherlands; 2 Department of Neurology, Rijnstate Hospital, Arnhem, the Netherlands; 3 Neurology Unit, Department of Medical, Surgical and Neurological Sciences, University Hospital of Siena, Siena, Italy; 4 Rheumatology Unit, Department of Medical, Surgical and Neurological Sciences, University Hospital of Siena, Siena, Italy; 5 Department of Neurology, Canisius-Wilhelmina Hospital, Nijmegen, the Netherlands; 6 Department of Neurosurgery, Radboud University Medical Center, Nijmegen, the Netherlands; 7 Department of Neurosurgery, Canisius Wilhelmina Hospital, Nijmegen, the Netherlands

**Keywords:** carpal tunnel syndrome, median nerve, ultrasonography, upper limit of normal, wrist circumference

## Abstract

**Background:**

Considerable between-center variability exists in the upper limit of normal (ULN) for the cross-sectional area (CSA) of the median nerve (MN) used to support the diagnosis of carpal tunnel syndrome (CTS).

**Objective:**

To evaluate whether a Dutch wrist circumference–dependent (WCD) equation for the ULN of MN CSA provides comparable classification of abnormal CSA in Italian and Dutch CTS populations.

**Methods:**

Wrist circumference (WC) and MN CSA were measured in 55 Italian (85 wrists) and 175 Dutch (175 wrists) patients with CTS. Abnormal wrists were identified using three thresholds: ULN9 (9 mm ^2^ ), ULN11 (11 mm ^2^ ), and a WCD equation ( *y* = 0.88 * *x* − 4, with *y* as ULN in mm ^2^ *x* as WC in cm). Findings were compared with electrodiagnostic results.

**Results:**

No significant differences in WC or MN CSA were observed between groups. The proportion of abnormal wrists in the Italian and Dutch populations, respectively, was 83.5 and 78.3% (ULN9, *p* = 0.321), 49.4 and 50.3% (ULN11, *p* = 0.895), and 61.1 and 55.4% (WCD, *p* = 0.379). Among 12 Italian patients with small wrists, abnormal results were found in 75.0% (ULN9), 25.0% (ULN11), and 66.7% (WCD). All Italian patients had abnormal electrodiagnostic testing results, compared with 66.3% of Dutch patients ( *p* < 0.0001).

**Conclusion:**

The WCD ULN for MN CSA yielded similar results in Italian and Dutch patients with CTS, and may be particularly useful in individuals with smaller wrists.

## Introduction

Carpal tunnel syndrome (CTS) is the most commonly encountered entrapment neuropathy, resulting from compression of the median nerve (MN) as it traverses the carpal tunnel. 11 Despite its prevalence, there is no universally accepted gold standard for its clinical diagnosis. In practice and research settings, diagnoses are frequently supported by electrodiagnostic testing (EDX) or ultrasonography. 11 22

In the Netherlands, ultrasonography is recommended as a first-line diagnostic tool due to its accessibility and noninvasive nature. 33 A range of ultrasonography parameters have been proposed for confirming CTS, with an increase in the cross-sectional area (CSA) of the MN at the wrist being most widely adopted. 44 55 However, no consensus exists regarding the upper limit of normal (ULN) for the CSA, with reported values varying considerably. One post hoc analysis, for instance, suggested a range of 9 to 15 mm ^2^ . 66

Previous research has demonstrated a strong correlation between the MN CSA and wrist circumference (WC), while other anthropometric variables did not independently correlate with CSA. 77 Additionally, WC has been shown to correlate positively with both weight and height. 88 Notably, the average height of the Dutch population (self-reported, 20 years or older) is the highest globally, with individuals born in 2001 averaging 169.3 cm for women and 182.9 cm for men. 99 In contrast, the average height in the Italian population is shorter at 162.6 cm for women and 176.5 cm for men. 1010 If height indeed influences WC and, by extension, the CSA of the MN at the wrist, this could introduce inter-population differences in CTS diagnosis.

The aim of this study was to compare the performance of a fixed and WCD ULN for MN CSA in Dutch and Italian patients with CTS. Rather than evaluating ultrasonography as a stand-alone diagnostic test, we sought to determine whether a WCD threshold yields comparable classification of abnormal MN CSA across populations with differing anthropometric characteristics. We hypothesized that, because WC may vary between populations, the WCD equation would account for these differences and result in similar proportions of abnormal ultrasonography findings across cohorts.

## Methods

This multicenter observational study included patients with CTS from Siena University Hospital (Italy) and from three centers in the Netherlands (Canisius–Wilhelmina Hospital, Zuyderland Hospital, and Radboud University Medical Center). 1111 Written informed consent was obtained from all participants, and ethical approval was granted by the local Medical Ethics Committee.

### Study Design

This multicenter observational cohort study compared ultrasonography findings between Italian and Dutch patients with CTS. The Italian cohort consisted of prospectively enrolled patients in whom ultrasonography and EDX testing were performed as part of routine diagnostic work-up.

The Dutch cohort comprised retrospectively identified patients from three centers in whom ultrasonography had previously been performed as part of standard clinical care. In the Netherlands, ultrasonography is commonly used as a first-line diagnostic test for CTS; therefore, EDX studies were not performed in all patients.

### Study Population

Patients aged 18 years and older with clinically suspected CTS were eligible for inclusion. Inclusion required the presence of pain and/or paraesthesia in the MN distribution, in addition to at least two of the following clinical features: (1) paraesthesia relieved by shaking the hand (positive Flick sign), (2) symptom exacerbation during certain activities (e.g., driving, cycling, phone use, reading); and (3) nocturnal paraesthesia. Exclusion criteria comprised a history or clinical signs of polyneuropathy, hereditary neuropathy with liability to pressure palsies, prior carpal tunnel release surgery, prior wrist trauma or surgery, rheumatoid arthritis, wrist arthrosis, diabetes mellitus, thyroid disease, alcoholism, and the presence of bifid MNs. Corticosteroid injections were not performed prior to diagnostic ultrasound at the participating centers, and ultrasonographic imaging was consistently obtained before any surgical intervention.

### Anthropometric Measurements

Height and weight were measured in all participants. WC was measured in both centers at the level of the distal wrist crease using a flexible plastic measuring tape with the wrist in neutral position and without compression of soft tissue, following an identical protocol.

### Ultrasound and Electrodiagnostic Studies

All participants underwent ultrasonography and EDX evaluation in accordance with previously published protocols. 1212 1313 In Italy, patients were enrolled if clinical symptoms were supported by EDX. Dutch participants were included based on clinical diagnosis alone, irrespective of EDX findings. Ultrasonography examinations were performed by experienced neurophysiology technicians in the Netherlands and by an experienced rheumatologist sonographer in Siena. The MN CSA at the carpal tunnel was outlined using the inner margin of the hyperechoic rim. CSA values were automatically calculated using the software integrated into the ultrasound systems. In the Netherlands, a Hitachi Aloka Arietta 850 system (Hitachi Aloka Medical Ltd., Tokyo, Japan) was used with a 5 to 17 MHz linear array transducer. In Siena, a MyLab X8 eXP (Esaote S.p.A., Genoa, Italy) was used, with 4 to 15 and 8 to 24 linear probes. All ultrasound measurements analyzed in this study were acquired during routine clinical evaluation prior to treatment and were not repeated specifically for research purposes.

### CSA Thresholds and Comparison

We compared the number of wrists in which CTS was confirmed using both fixed and WCD ULN values for CSA. The fixed ULN thresholds were > 9 mm ^2^ (ULN9) in the Italian center 1414 and > 11 mm ^2^ (ULN11) in the Dutch centers. 1515 The WCD ULN was calculated using the formula *y* = 0.88 * *x* − 4, where *y* represents the ULN of the CSA in mm ^2^ and *x* the WC in centimeters, as previously described. 77 These findings were then compared to the results of EDX studies.

### Statistics Analysis

Statistical analyses were conducted using SPSS Statistics version 26.0 (IBM, Armonk, New York, United States). Data distribution was assessed using Q–Q plots, histograms, and the Kolmogorov–Smirnov test. Categorical variables were analyzed using the chi-square test, and continuous variables with a nonnormal distribution were evaluated using the Mann–Whitney test. A *p* -value of < 0.05 was considered statistically significant.

## Results

Table 1Table 1 presents the biometric characteristics of the study population. A total of 260 wrists from 230 patients with CTS were included. Patients enrolled in Italy were significantly older, whereas those included in the Netherlands were taller and heavier (small effect sizes *r* = 0.14–0.19). No significant differences were observed between groups with respect to sex, median WC, or median MN CSA. However, the median duration of symptoms was significantly longer among the Italian participants (moderate effect size *r* = 0.38).

**Table 1:** **Table 1** Patient characteristics

	Patients with CTS in Italy	Patients with CTS in the Netherlands	*p* -Value	Effect size *r*
Participants	55	175		
Women/men	36/19	122/53	0.552 a	–
Median age, y (IQR)	64 (17)	55 (22)	0.005 b	0.19
Left/right/bilateral wrist	8/17/30	83/92/0	*<* 0.0001 a	–
Median height, cm (IQR)	165 (12)	168 (10)	0.039 b	0.14
Median weight, kg (IQR)	70.0 (18.0)	78.0 (21.5)	0.025 b	0.15
Median BMI (IQR)	26.0 (5.3)	27.4 (5.8)	0.168 b	0.09
Median wrist circumference, cm (IQR)	17.0 (1.8)	17.0 (1.8)	0.328 b	0.06
Median CSA of MN, mm ^2^ (IQR)	11.0 (4.2)	11.1 (4.3)	0.614 b	0.03
Median duration of symptoms, mo (IQR)	24 (24)	6 (22)	*<* 0.001 b	0.38

Abbreviations **:** CSA, cross-sectional area; CTS, carpal tunnel syndrome; MN, median nerve.

Note: Effect size *r* is calculated as Z/√N for the Mann–Whitney U test comparisons of continuous variables. Not applicable for categorical variables.

a Chi-square test.

b Mann–Whitney U Test.

Table 2Table 2 summarizes ultrasonographic and electrodiagnostic findings. The proportion of wrists classified as abnormal based on ultrasonography was comparable between the Italian and Dutch cohorts, including when the WCD threshold was applied. When using the lowest ULN (the fixed threshold applied in Italy), the majority of ultrasonography results were deemed abnormal. The proportion of abnormal wrists using the WCD ULN was similar between cohorts (61.1% vs. 55.4%; risk difference 5.7%, 95% CI −7.7 to 19.1). Because Italian patients were enrolled only if electrodiagnostically confirmed, an additional sensitivity analysis restricted to EDX-positive wrists was performed ( Table 3Table 3 ). Under these conditions, results remained comparable between cohorts for the WCD ULN (61.1% vs. 69.8%; risk difference −8.7%, 95% CI −22.0 to 4.6), whereas application of ULN11 revealed a significant between-cohort difference.

**Table 2:** **Table 2** Results of the different ultrasound ULN and EDX studies

Sonography	ULN	Italy	The Netherlands	Total	*p* -Value
Total		85	175	260	
Abnormal, as defined by					
	ULN9 (9 mm ^2^ )	71 (83.5%)	137 (78.3%)	208 (80.0%)	0.321 a
	ULN11 (11 mm ^2^ )	42 (49.4%)	88 (50.3%)	130 (50.0%)	0.895 a
	WCD ULN	52 (61.1%)	97 (55.4%)	149 (57.3%)	0.379 a
Abnormal based on EDX					
		85 (100%)	116 (66.3%)	203 (78.1%)	<0.0001 a

Abbreviations **:** EDX, electrodiagnostic testing; ULN, upper limit of normal; ULN11, fixed ULN of 11 mm ^2^ (Dutch cohort); ULN9, fixed ULN of 9 mm ^2^ (Italian cohort); WCD ULN, wrist circumference–dependent ULN, calculated as y = 0.88 *x* − 4, where *y* is the ULN in mm ^2^ and *x* is wrist circumference in cm.

a Chi-square test.

**Table 3:** **Table 3** Ultrasonographic classification of CTS in Italian and Dutch cohorts restricted to EDX confirmed wrists

Sonography	ULN	Italy	The Netherlands	Total	*p* -Value
Total		85	116	201	
Abnormal, as defined by					
	ULN9 (9 mm ^2^ )	71 (83.5%)	102 (87.9%)	173 (86.0%)	0.373 a
	ULN11 (11 mm ^2^ )	42 (49.4%)	74 (63.8%)	116 (57.7%)	0.041 a
	WCD ULN	52 (61.1%)	81 (69.8%)	133 (66.2%)	0.200 a

Abbreviations **:** CTS, carpal tunnel syndrome; EDX, electrodiagnostic testing; ULN, upper limit of normal; ULN9, fixed ULN of 9 mm ^2^ (Italian cohort); ULN11, fixed ULN of 11 mm ^2^ (Dutch cohort); WCD ULN, wrist circumference–dependent ULN, calculated as y = 0.88 *x* − 4, where *y* is the ULN in mm ^2^ and *x* is wrist circumference in cm.

a Chi-square test.

To further explore the relatively lower classification rate using the WCD ULN, relationships between MN CSA and age, symptom duration, and WC were examined. CSA showed no meaningful correlation with age ( *r* = 0.051) and only a weak correlation with symptom duration ( *r* = 0.162). In contrast, visualization of CSA values relative to the WCD ULN demonstrated that a substantial proportion of clinically suspected, EDX-negative wrists fell below the WCD threshold; 43 wrists (72.9%) did not exceed the WCD ULN ( Supplementary Fig. S1 , available in the online version only). When analysis was restricted to EDX-confirmed wrists, the distribution of CSA relative to the WCD ULN did not differ significantly between Italian and Dutch cohorts (Mann–Whitney U test, *p* = 0.096).

**Fig. 1:**
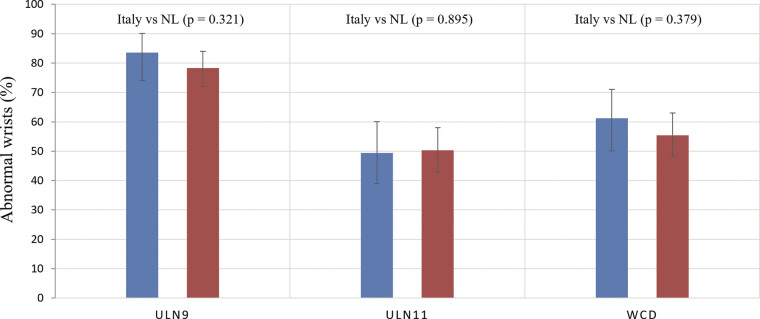
**Fig. 1** Proportion of wrists classified as abnormal by ultrasonography in Italian and Dutch cohorts using three upper limits of normal (ULN) for the cross-sectional area (CSA) of the median nerve (MN). ULN refers to a fixed ULN of 9 mm ^2^ , ULN11 to a fixed ULN of 11 mm ^2^ , and WCD ULN to a wrist circumference–dependent threshold using the formula *y* = 0.88 ^*^ x − 4 ( *y* = ULN of the CSA of the MN in mm ^2^ and *x* = wrist circumference in cm). Bars represent percentages with 95% confidence intervals. *p* -Values indicate between-cohort comparisons for each threshold ( *χ* ^2^ test).

Fig. 1Fig. 1 illustrates the number of abnormal ultrasonography results in both populations, stratified by the different ULN thresholds.

Number of wrists with CTS confirmed by ultrasonography and EDX studies in Italian and Dutch participants. The fixed ULN for the CSA of the MN was defined as > 9 mm ^2^ (ULN9) in the Italian cohort and > 11 mm ^2^ (ULN11) in the Dutch cohort. The WC–dependent ULN (WCD ULN) was calculated using the formula *y* = 0.88 * *x* − 4 ( *y* = ULN of the CSA of the MN in mm ^2^ and *x* = WC in cm). ( Table 2Table 2 )

Ultrasonographic classification of CTS in Italian and Dutch cohorts restricted to electrodiagnostically confirmed wrists, using fixed and WCD ULN for MN CSA. ( Table 3Table 3 )

Table 4Table 4 presents the ultrasonography findings for a subset of Italian participants ( *n* = 12) with WCs ranging between 14.6 cm (−2 standard deviation) to 15.8 cm (−1 standard deviation).

**Table 4:** **Table 4** Ultrasound classification of wrists with small wrist circumference in the Italian cohort

Sonography		Number of wrists with CTS in Italian patients (% of wrists)
Total		85
	Upper limit of normal (ULN)	
Patients with a small wrist circumference		12/85 (14.1%)
Abnormal, as defined by		
	ULN9	9/12 (75%)
	ULN11	3/12 (25%)
	WCD ULN	8/12 (66.7%)

Abbreviations **:** CTS, carpal tunnel syndrome; ULN9, fixed ULN of 9 mm ^2^ (Italian cohort); ULN11, fixed ULN of 11 mm ^2^ (Dutch cohort); WCD ULN, wrist circumference–dependent ULN, calculated as *y* = 0.88 *x* − 4, where *y* is the ULN in mm ^2^ and *x* is wrist circumference in cm.

Sonographic detection of CTS in Italian patients with WCs between 14.6 cm (−2 standard deviation) and 15.8 cm (−1 standard deviation), using three different upper limits of normal (ULNs) for the CSA of the MN: a fixed ULN of 9 mm ^2^ (ULN9), a fixed ULN of 11 mm ^2^ (ULN11), and a WCD ULN calculated using the formula *y* = 0.88 * *x* − 4 (with *y* representing the ULN of the MN CSA in mm ^2^ and *x* the WC in cm). ( Table 4Table 4 )

## Discussion

This study found that a Dutch WCD ULN for MN CSA produced similar results in the Italian and Dutch cohorts, although the overall proportion of wrists exceeding the WCD threshold was lower than expected. As expected, the fixed ULN of 9 mm ^2^ , used in the Italian center, yielded the highest classification rate.

We hypothesized that, despite anticipated differences in WC and MN CSA between the populations, the WCD equation would offer comparable proportions of abnormal MN CSAs. Interestingly, no significant differences in these anthropometric features were observed. WC was measured using an identical protocol in both centers, making systematic measurement differences unlikely. Although Dutch participants were slightly taller on average, this modest difference did not translate into differences in WC and is unlikely to be clinically relevant. This small difference in height may be partially attributed to regional variation: our Dutch data were collected from the southern provinces, where the average height is lower than in the north. 99 Participants from Italy were recruited in Tuscany, a region with relatively taller individuals compared to other Italian regions. 1616 Historical gene flow from southern Europe into the southern Netherlands may also have contributed to the unexpectedly similar biometric profiles. 1717 Anthropometric variation between the symptomatic CTS populations appeared limited. The comparable wrist dimensions between cohorts may help explain the similar behavior of the WCD approach across centers.

While normative data for MN CSA have been widely published, few studies have explicitly examined population-level differences. 44 In one study, MN CSA remained significantly different between Indian and Dutch healthy participants even after adjusting for age, height, and weight. 1818 A systematic review similarly reported inter-ethnic variability, although most studies fail to document participant ethnicity. 1919 Another study comparing American and Italian CTS cohorts found comparable MN CSA values at the forearm, but a higher wrist-to-forearm ratio in the ultrasonography cohort due to a larger CSA at the wrist. 2020 In contrast, our findings revealed similar MN CSA and WC in both symptomatic CTS cohorts.

A notable and unexpected finding was the lower proportion of wrists exceeding the WCD ULN compared with earlier validation studies. 2121 2222 2323 2424 2525 2626 Additional analyses suggest that this reflects differences in disease spectrum rather than failure of the method itself. Although the current study population was older and symptom duration was longer in the Italian cohort, age and symptom duration showed only weak associations with MN CSA and therefore do not completely explain the observed findings. Visualization of CSA values relative to the WCD threshold demonstrated that many clinically suspected but electrodiagnostically negative wrists fell below the threshold, while CSA distributions were otherwise similar across cohorts. In this subgroup, diagnostic misclassification of CTS cannot be excluded. When analyses were restricted to the EDX-confirmed wrists, the distribution of CSA relative to the WCD threshold did not differ significantly between Italian and Dutch patients, supporting comparable behavior of the WCD approach across populations when disease definition is harmonized.

Alternative approaches to enhance diagnostic performance include the use of disease-specific thresholds and within-patient comparisons, as routinely applied in nerve conduction studies. 2727 Metrics such as the WFR and nerve/tendon ratio have shown promise as anthropometric-independent indices for CTS diagnosis. 1414 2828

We explored the utility of the WCD approach in a subset of Italian patients ( *n* = 12) with particularly small wrists (WC between 14.6 cm [−2 standard deviation] and 15.8 cm [−1 standard deviation]). In this group, 66.7% were classified as abnormal using the WCD formula, 75.0% using ULN9, and only 25.0% using ULN11. This supports previous observations that the WCD method may be particularly valuable in patients with small wrist dimensions. 1111

This study has several limitations. First, as both cohorts consisted of symptomatic patients, the present study was not designed to derive or validate population-specific normative ULN values, but rather to evaluate the performance of an existing WCD threshold across CTS populations. Second, the longer symptom duration and older age of Italian participants may have affected CSA measurements. Third, not all Dutch patients had EDX confirmation, potentially introducing diagnostic heterogeneity. Lastly, differences in ultrasound equipment and operator experience between centers may have contributed to variability, although scans were performed by experienced personnel using established protocols. An expert panel advised that EDX studies should be performed in individuals aged over 70. 2929 MN CSA measurement by ultrasonography has demonstrated good inter- and intra-rater reliability in previous studies, 1212 3030 with measurement variability small relative to commonly used diagnostic thresholds. Because the present analyses focus on the proportion of wrists exceeding predefined CSA thresholds rather than very small absolute CSA differences, it is unlikely that measurement variability meaningfully influenced the between-cohort comparisons.

## Conclusion

This study, though hampered by the limitations discussed above, demonstrates that a Dutch-derived WCD formula for determining the ULN of MN CSA at the carpal tunnel yields comparable results in Italian and Dutch patients with CTS. No significant differences in WC or MN CSA were observed between the two populations. The WCD approach may offer particular value in individuals with smaller wrist dimensions. These findings suggest that WCD thresholds may be less influenced by population-specific factors than fixed cut-offs.
