# Supplementary material for: Wrist Circumference–Dependent Thresholds for the Median Nerve in Carpal Tunnel Syndrome: A Cross-Cohort Comparison
Source: J Brachial Plex Peripher Nerve Inj. 2026 Apr 30;21(1):e7–e12. doi: 10.1055/a-2837-4022 (PMC13132619; doi:10.1055/a-2837-4022)
Supplement: Supplementary file 1 — Supplementary Material [file 10-1055-a-2837-4022_28681380.pdf]

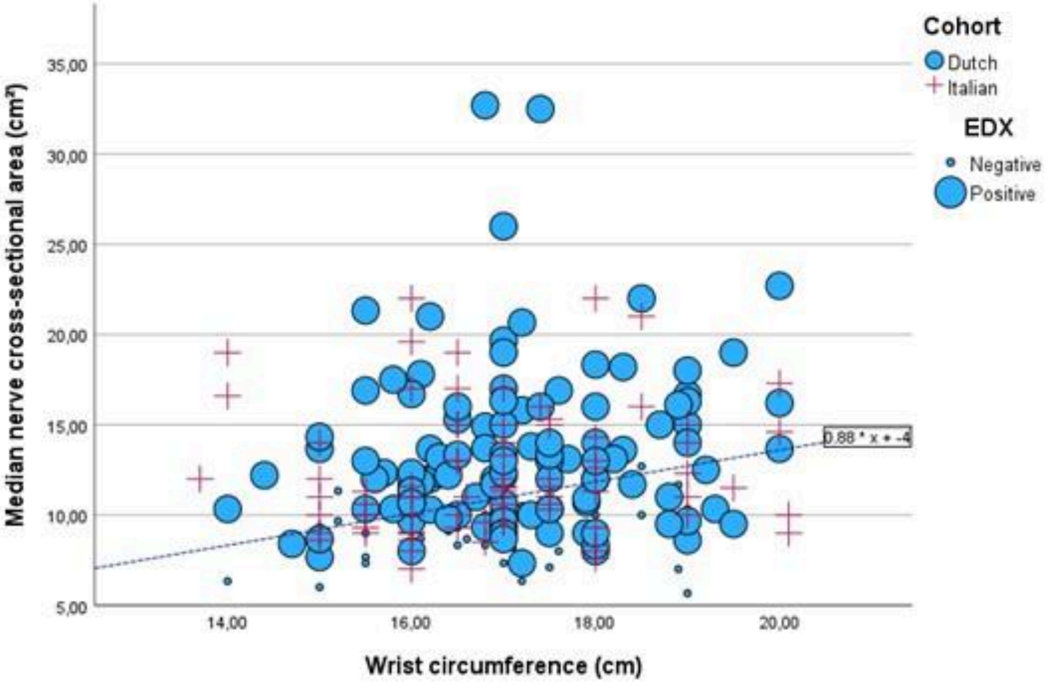

Supplementary Fig. S1 Scatterplot of median nerve cross-sectional area versus wrist circumference in Italian and Dutch participants, with the wrist circumference-dependent upper limit of normal (WCD ULN) indicated by the dotted line. Dutch patients are represented by blue circles and Italian patients by red crosses; larger blue circles indicate EDX-positive wrists and smaller blue circles EDX-negative wrists. Clinically suspected, EDX-negative wrists largely fall below the WCD threshold, whereas in EDX-positive CTS, the distribution of CSA values relative to the WCD ULN is comparable between cohorts.
